# Bi-Directional Relationship Between Bile Acids (BAs) and Gut Microbiota (GM): UDCA/TUDCA, Probiotics, and Dietary Interventions in Elderly People

**DOI:** 10.3390/ijms26041759

**Published:** 2025-02-19

**Authors:** Emanuele Francini, Gretta V. Badillo Pazmay, Stefania Fumarola, Antonio Domenico Procopio, Fabiola Olivieri, Francesca Marchegiani

**Affiliations:** 1 Clinic of Laboratory and Precision Medicine, IRCCS INRCA, 60121 Ancona, Italy; e.francini@inrca.it (E.F.); a.d.procopio@univpm.it (A.D.P.); 2 Advanced Technology Center for Aging Research, IRCCS INRCA, 60121 Ancona, Italy; g.badillo@inrca.it (G.V.B.P.); s.fumarola@inrca.it (S.F.); f.olivieri@univpm.it (F.O.); 3 Laboratory of Experimental Pathology, Department of Clinical and Molecular Sciences, Università Politecnica delle Marche, 60100 Ancona, Italy

**Keywords:** bile acids (BAs), dysbiosis, gut microbiota (GM), elderly, probiotics supplementation, UDCA/TUDCA supplementation

## Abstract

The gut microbiota (GM), the set of microorganisms that colonizes our intestinal tract, can undergo many changes, some of which are age related. Several studies have shown the importance of maintaining a healthy GM for a good quality of life. In the elderly, maintaining a good GM may become a real defense against infection by pathogens, such as *C. difficile*. In addition to the GM, bile acids (BAs) have been shown to provide an additional defense mechanism against the proliferation of pathogenic bacteria and to regulate bacterial colonization of the gut. BAs are molecules produced in the host liver and secreted with the bile into the digestive tract, and they are necessary for the digestion of dietary lipids. In the gut, host-produced BAs are metabolized by commensal bacteria to secondary BAs. In general GM and host organisms interact in many ways. This review examines the relationship between GM, BAs, aging, and possible new approaches such as dietary interventions, administration of ursodesoxycholic acid/tauroursodesoxycholic acid (UDCA/TUDCA), and probiotics to enrich the microbial consortia of the GM in the elderly and achieve a eubiotic state necessary for maintaining good health. The presence of Firmicutes and Actinobacteria together with adequate levels of secondary BAs would provide protection and improve the frailty state in the elderly. In fact, an increase in secondary BAs has been observed in centenarians who have reached old age without serious health issues, which may justify their active role in achieving longevity.

## 1. Introduction

Gut microbiota (GM) is the collection of microorganisms (bacteria, viruses, and fungi) that have colonized the intestinal tract. The GM of a healthy human adult varies between individuals and contains an average of 150 bacterial species, 95% of which belong to the phyla Firmicutes and Bacteroidetes and the remaining 5% to the phyla Actinobacteria, Proteobacteria, Tenericutes, Verrucomicrobia, and Fusobacteria, with a Firmicutes/Bacteroidetes ratio (F/B ratio) of approximately 0.8 to 1 [1,2,3]. The composition of GM is influenced by intestinal pH [4], diet [5], low fiber intake [6], health, drugs/supplements, pathological status [7], slow intestinal transit, bowel constipation [8,9], and geographical origin [10]. Also, the GM changes with age [11]. The importance of a healthy GM for promoting a good quality of life has been demonstrated in several studies that have evaluated the health status of older people in relation to diet [12], physical fitness [13], and metabolome [14,15]. An altered GM can lead to dysbiosis and intestinal problems, such as diarrhea and irritable bowel syndrome (IBS), or problems associated with cardiovascular, metabolic, liver, and neurodegenerative diseases [16,17,18,19,20]. It has been reported that the GM of older people is characterized by reduced biodiversity, resulting in decreased Firmicutes and increased levels of Bacteroidetes and Proteobacteria [11,21,22,23,24]. Decreasing Firmicutes and increasing Bacteroidetes decreases the F/B ratio, which may be a potential indicator of dysbiosis [24,25]. A decrease in *Bifidobacteria* (Actinobacteria) was also observed in elderly people [24,26]. Salosensaari et al. have reported a strong correlation between mortality and the prevalence of Proteobacteria (Enterobacteriaceae) in the GM [27]. Interestingly, also in *Drosophila*, an expansion of the Gammaproteobacteria has been correlated with intestinal barrier dysfunction and organismal death [28]. An increase in Proteobacteria, Enterobacteriales, and Pseudomonadales (Gammaproteobacteria class) was also observed in the GM of progeroid mice with premature aging and increased mortality at 4 months of age compared to the wild type [29]. In addition to low Firmicutes, lower levels of GM short-chain fatty acids (SCFAs) like butyrate, acetate, and propionate were observed in older people [12]. Butyrate is generally associated with gut health, and its deficiency has been linked to deficits in intestinal permeability and intestinal barrier fragility [30]. The interaction between the host genome and the gut microbiome can influence and regulate several key metabolic pathways, including the metabolism of vitamins, enzymes, and bile acids (BAs) [1,30,31,32,33,34,35].

BAs are among the most structurally diverse biomolecules in nature [32]. The main primary BAs produced in the human liver are cholic acid (CA) and chenodeoxycholic acid (CDCA) [36]. In the intestine, primary BAs enable the digestion of dietary lipids and have a bactericidal effect [32,37]. The relationship between BAs and GM is bidirectional; in the gut, BAs counteract the proliferation of pathogenic bacteria and regulate bacterial colonization, but their structure is affected by bacterial metabolism [32,35,38,39]. Pathways associated with the bacterial transformation of BAs by the GM include the reactions of deconjugation, dehydrogenation, and dehydroxylation [30,36]. The deconjugation reaction is mediated by bacteria with the enzyme bile salt hydrolase (BSH), occurs predominantly in the small intestine, and produces deconjugated primary BAs [35,40]. In the colon, bacteria with the enzyme 7α-dehydroxylase convert deconjugated primary BAs into secondary BAs, such as deoxycholic (DCA) and lithocholic acid (LCA) [30,32,39]. DCA and LCA are hydrophobic molecules that are less capable of supporting the digestion of dietary lipids but still have an important role in immunity, serotonin production, cell signaling, and preventing *Clostridioides difficile* infection [32]; however, high concentrations of DCA and LCA can have cytotoxic effects and cause damage to colonocyte membranes, trigger oxidative stress, induce intestinal dysbiosis, and contribute to carcinogenesis processes in the colon [41,42,43]. The GM can synthesize many other BAs, which are discussed in detail below [44]. In addition to their digestive function, BAs may also play a role in signaling pathways triggered by their binding to bile receptors, including the nuclear farnesoid X receptor (FXR), the pregnane X receptor (PXR), and the G protein-coupled membrane receptor (GPCR) known as TGR5 [45]. Secondary (hydrophobic) BAs have a higher affinity for these receptors than primary (hydrophilic) BAs [32]. In general, BAs have contrasting roles, with both positive and negative effects on humans, related to the variation of their normal circulating levels [32]. Diet, antibiotics, and probiotics are some factors that can affect circulating levels of secondary BAs [30,46,47,48]. Changes in secondary BA levels have been observed in obesity, metabolic and cardiovascular disease, and other age-related problems, such as gallstones [49]. The incidence of gallstones increases with age due to increased gallbladder dysfunction and associated secondary complications such as cholecystitis, cholangitis, and pancreatitis. In addition, acute cholecystitis caused by cystic duct obstruction and bacterial infection can progress to sepsis and multi-organ dysfunction syndrome (MODS) [50]. Different types of dysbiosis are other factors that can influence the BA pool by altering deconjugated and secondary BA levels, with consequences for the onset of metabolic disorders and susceptibility to infection (e.g., *C. difficile*) [24,51]. When bacteria with BSH activity are reduced in the GM, deconjugated and secondary BAs decrease accordingly, which can lead to metabolic disorders associated with altered FXR and TGR5 receptor activity and a higher susceptibility to infections [32,49,51]. The decrease in Firmicutes may cause a subsequent decrease in BSH activity with effects similar to those described above [11,21,22,23,24,38,52]. In the elderly, re-establishing an eubiotic status together with normal levels of Firmicutes and Actinobacteria through dietary intervention and/or the administration of probiotics and/or UDCA/TUDCA may be able to increase the BSH activity [49].

The aim of this review is to investigate the role of BAs and the mechanisms by which we can intervene to restore optimal BSH enzyme levels in the elderly.

## 2. Bile Acid Metabolism

### 2.1. Bile

Bile is composed of 95% water and many inorganic and organic components such as BAs, cholesterol, sodium, potassium, and phospholipids with a pH of 7.5–8.0 [53]. Bile helps emulsify and dissolve dietary fats to enable the absorption of dietary lipids and fat-soluble vitamins [54]. It also has an antibacterial effect, preventing the excessive growth of bacteria in the intestines due to the BAs it contains. In addition, bile participates in the host’s innate immune defense system, allowing the excretion of endogenous substances, such as bilirubin and excess cholesterol, and exogenous substances, such as xenobiotics and heavy metals [38,55].

Bile is a difficult fluid to analyze, and much information about it comes from studies in animal models (e.g., mice) in vitro or in vivo in cholestatic subjects [55]. Bile is produced in the liver (600 mL to 1.2 lt per day) within the hepatocytes.

With age, the bile ducts undergo anatomical and physiological changes, and geriatric patients tend to present with mild symptoms of biliary disease, which can progress to acute conditions if left untreated. In addition, in older people with acute cholecystitis, the symptoms may be atypical and the physical signs may be masked by the neuropathy [56]. Finally, the true reading of the white blood cell (WBC) count can be obscured by age, so a more thorough examination would be appropriate in cases where gallstones are suspected [56].

### 2.2. Bile Acids

**Figure 1:**
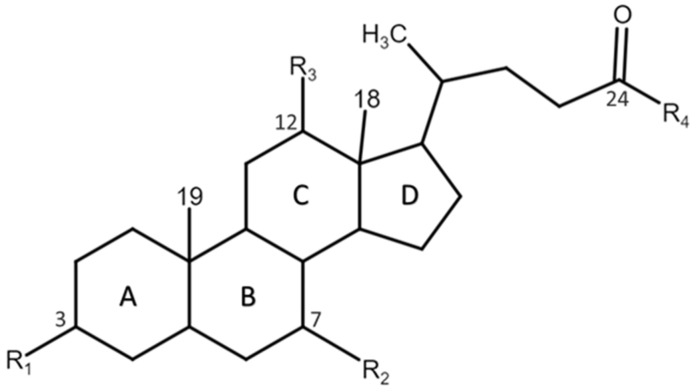
Chemical structure of human BAs. Lettering and atom numbering of the BA skeleton. The four rings A–D form a sterane core showing the relationship between BA pools.

BAs are steroid acids produced in the liver by the removal of the last three carbon atoms of the aliphatic side chain of cholesterol [54]. Human BAs have 24 carbon atoms, and their general structure is characterized by a steroid nucleus consisting of four cyclic rings (A, B, C, D), hydroxyl groups (-OH) attached to the steroid nucleus, which can be in positions 3, 7, and 12, and a side chain attached at position C17 with a carboxyl group (-COOH) at the end, which can be conjugated to glycine and taurine (see Figure 1). The different positions of the -OH groups (3, 7, and 12) give these molecules a significantly higher polarity than cholesterol [57].

The structure of BAs varies with respect to side chain length (C27/C24), side chain carboxylation (BAs) or hydroxylation (bile alcohols), side chain conjugation (amino acids or sulfate), cis stereochemistry of the A/B ring (5β-H or non-planar ring system) or trans (5α-H or planar ring system), and hydroxylation patterns and the stereochemistry of the hydroxyls (α, β, Ω) [32].

**Figure 2:**
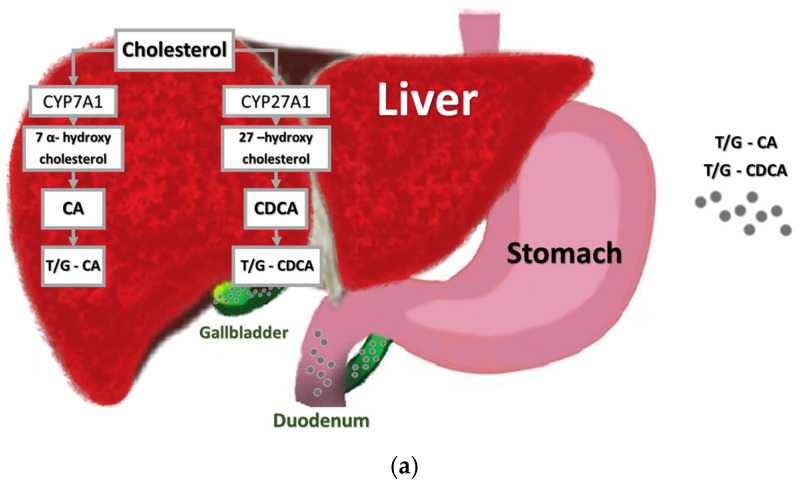

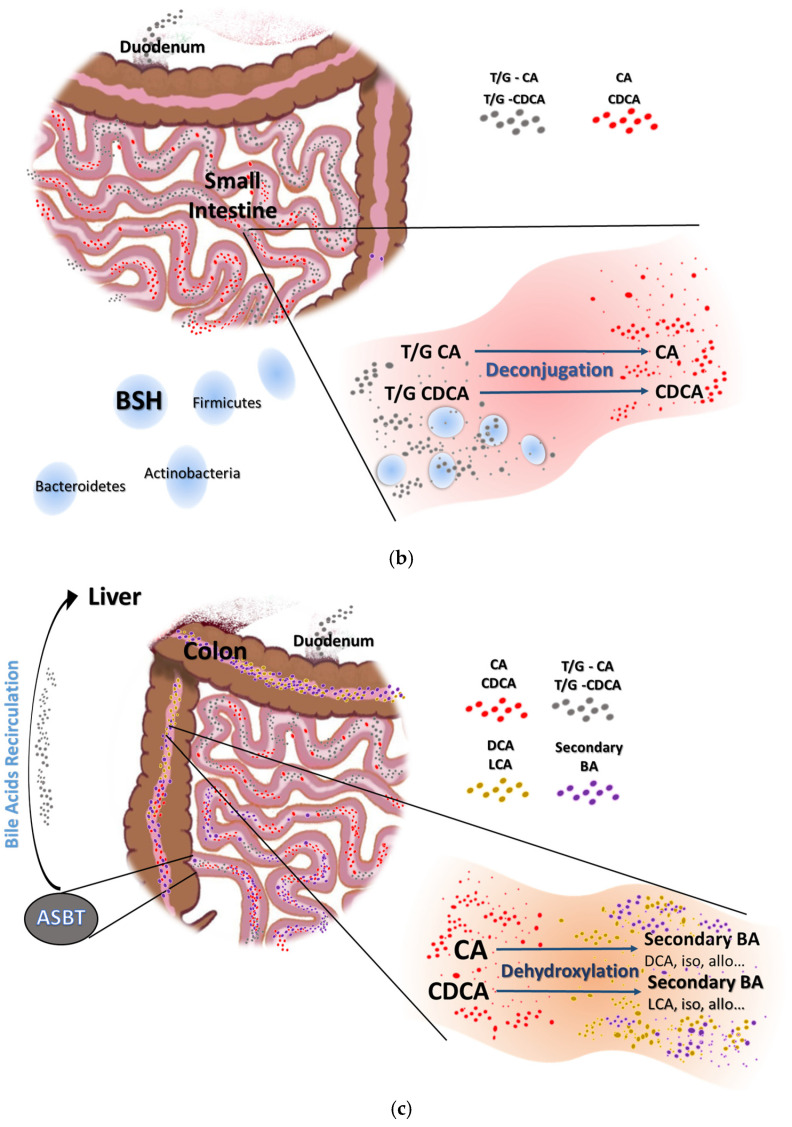
Overview of bile acid metabolism and enterohepatic recirculation. (**a**) Primary BAs, such as CA and CDCA, in humans are synthesized by hepatocytes and then conjugated with either taurine or glycine (gray circles). Conjugated BAs are then secreted into the bile and stored in the gallbladder until secreted in the duodenum. (**b**) The deconjugation reaction of T/G-CA and T/G-CDCA in CA and CDCA (red circles) is mediated by BSH bacteria and occurs predominantly in the small intestine. (**c**) Deconjugated primary BAs reduce absorption through ASBT, which serves to transport conjugated BAs from the small intestine into portal recirculation and then into the hepatocyte. Instead, the bacteria in the colon convert deconjugated primary BAs into DCA, LCA (yellow circles), and other secondary BAs (purple circles). Abbreviations: BAs, bile acids; CA, cholic acid; CDCA, chenodeoxycholic acid; DCA, deoxycholic acid; LCA, lithocholic acid; ASBT, Apical Sodium-dependent Bile Acid Transporter; BSH, bile salt hydrolase.

The hepatic biosynthesis of BAs produces amphipathic molecules with two distinct regions: a hydrophilic (polar) region, given by the hydroxyl (-OH) and carboxyl (-COOH) groups, and a lipophilic (apolar) region, given by the steroid nucleus; this gives BAs their detergent properties. The structure of BAs allows them to form micelles in an aqueous solution. Micelles are small aggregates of molecules in which the polar groups are on the outside and the apolar groups are on the inside, and they are capable of accommodating water-insoluble lipid molecules [54]. Micelles play an important role in the digestion of dietary lipids such as phospholipids, fatty acids, cholesterol, and fat-soluble vitamins [32,54]. The liver is the only organ that contains the enzymes required for de novo biosynthesis of BAs from cholesterol [32]. In humans, BA synthesis is regulated by a negative feedback pathway mediated by FXR [58]. Binding between BAs and FXR induces the expression of fibroblast growth factor (FGF15/19), which is released into the portal circulation, reaches the liver, and binds to hepatocyte surface receptors, limiting BA synthesis by inhibiting the activity of the enzyme 7α-hydroxylase (CYP7A1) [30]. The main BAs produced in humans are CA and CDCA; there are two metabolic pathways of BA biosynthesis: the classic/neutral pathway and the alternative/acidic pathway, both of which are extensively described in the review by Russel et al. [59] (see Figure 2a).

Briefly, primary fatty acids are synthesized from cholesterol in hepatocytes via two pathways, classical and alternative (Figure 2a); most conversion follows the classical (neutral) pathway, in which cholesterol is first converted to 7α-hydroxycholesterol by the enzyme cholesterol 7α-hydroxylase (CYP7A1). Subsequently, the enzyme 3β-hydroxy-Δ5-C27-steroid dehydrogenase/isomerase reduces 7α-hydroxycholesterol to 7α-hydroxy-4-cholesten-3-one. The final product is formed by the action of the enzyme sterol 12α-hydroxylase (CYP8B1) on 7α-hydroxy-4-cholesten-3-one to form chenodeoxycholic acid (CDCA) or/and cholic acid (CA) [60].

The alternative (acid) pathway leads to the conversion of cholesterol to 27-hydroxycholesterol via the enzyme 27α-hydroxylase (CYP27A1). A non-specific 7α-hydroxylase (CYP7B1) hydroxylates 3β-hydroxy-5-cholestenoic acid to 3β, 7α-dihydroxy-5-cholestenoic acid, followed by HSD3B1/3B2 to synthesize 7α-hydroxy-3-oxo-4-cholestenoic acid in the liver. This pathway produces more CDCA than CA [60].

BAs synthesized from cholesterol are immediately conjugated in the liver with the amino acids glycine and taurine and then excreted with the bile. Taurine is a sulfur-containing amino acid that is synthesized in the liver mainly from sulfur-containing amino acids, such as cysteine, and to a lesser extent from methionine, through a biosynthetic process involving several enzymatic steps [61]. Conjugation is a process that occurs within hepatocytes by BA-CoA-amino-N-acyltransferase (BAAT) [53]. In humans, the primary conjugated BAs are glycocholic acid (GCA), glycochenodesoxycholic acid (GCDCA), taurocholic acid (TCA), and taurochenodesoxycholic acid (TCDCA) [30]. High concentrations of BAs remain in the duodenum, jejunum, and proximal ileum to help digest and absorb lipids [34]. In the small and large intestines, bacteria modify BAs by deconjugation, epimerization, oxidation, and dehydroxylation reactions [34,36] (see Figure 2b,c).

Deconjugation is mediated by the bacterial enzyme BSH (see Figure 2b). BSH enables the hydrolysis of the N-acylamide C-24 bond that binds BA to its amino acid conjugates taurine and glycine [52]. BSH differs by subunit size and composition, pH optimal, kinetic properties, substrate specificity, gene organization, and regulation [34]. Deconjugated BAs, formed through deconjugation by BSH, are less efficient at solubilizing and absorbing lipids in the gut but have greater bactericidal activity [4,62].

**Figure 3:**
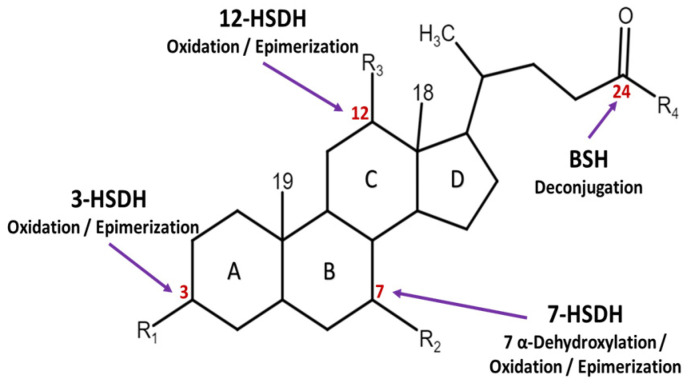
Biotransformation of BAs. The oxidation and epimerization on carbon 3, 7, and 12 positions (in red color) are determined by different microbial hydroxysteroid dehydrogenases (HSDHs), while the deconjugation of taurine and glycine on C-24 (in red color) is mediated by bile salt hydrolase (BSH).

The removal of -OH groups in C3, C7, and C12 of BAs is achieved by the 3α-dehydroxylation, 7α-dehydroxylation, and 12α-dehydroxylation reactions of BAs, expressed in BA inducible (bai) operon. The oxidation and epimerization of -OH groups (3, 7, 12) on BAs (see Figure 3) are determined by different microbial hydroxysteroid dehydrogenases (3α/β-, 7α/β-, 12α/β- HSDH) expressed in different genes with species-specific differences. HSDH pairs can reversibly epimerize steroids from α-hydroxy to β-hydroxy conformations (see Figure 2c). The set of bacterial genes involved in biotransforming BAs and sterols has been termed the “sterolbiome” [32]. Analysis of human fecal samples revealed more than 50 different secondary BAs, which are derived from the bacterial metabolism of primary BAs in the host [30,32]. A more detailed description of all the enzymes and bacteria involved in the transition from primary to secondary BAs was reported by Ridlon et al. [32,34,63].

In the human colon, bacterial 7β-HSDH converts small amounts of CDCA to ursodesoxycholic acid (UDCA) [30], and 7α-dehydroxylation (the Hyleon–Bjorkhem pathway) [63] converts CA, CDCA, and UDCA to DCA and LCA [32,34,64]. The 7α-dehydroxylation reaction increases the hydrophobicity of BAs, which directly correlates with their bactericidal activity by increasing their affinity for the phospholipid bilayer of bacterial cell membranes [4]. In humans, hepatic metabolism is not able to convert DCA and LCA into the respective primary BAs via the 7α-hydroxylation reaction, so they easily accumulate in the enterohepatic circulation, especially DCA [34]. Bacteria in the colon produce other BAs such as ursocholic acid (UCA), 12-epicholic acid (12-EPA), isocholic acid (ICA), isochenodeoxycholic acid (iCDCA), isoDCA (iDCA), isoLCA (iLCA), 3-oxo-LCA, allo-LCA, 3-oxoallo-LCA, and isoallo-LCA [30,32,44]. Secondary BAs can be reabsorbed through passive absorption in the intestinal wall or be excreted with the feces [32,54,57,64]. Secondary BAs that are absorbed by passive transport in the intestine and through portal circulation arrive at the liver where they are further modified in tertiary BAs [32]. In the liver, LCA is sulfated to 3-sulfo-LCA [32]. 3-sulfo-LCA is conjugated to glycine and taurine and is secreted with the bile in the duodenum and then can be deconjugated by bacterial BSH in the intestine. 3-sulfo-LCA can also be desulphurized by bacteria with the arylsulfatase enzyme, which removes the sulfate to form LCA [32]. A list of the human BAs is reported in Table 1.

### 2.3. Enterohepatic Circulation of Bile Acids

BAs synthesized in hepatocytes are secreted into the bile via the bile salt export pump (BSEP) [54]. During the interprandial phase, when the sphincter of Oddi is closed, bile flows into the gallbladder. In the intra- and post-prandial phases, the presence of the food bolus stimulates the contraction of the gallbladder to release bile into the duodenum. In the distal ileum, active transport via the ileal sodium/bile acid cotransporter (IBAT), found on the apical membrane of enterocytes, facilitates the absorption of conjugated BAs, which then enter the enterohepatic bile salt circulation [54].

**Figure 4:**
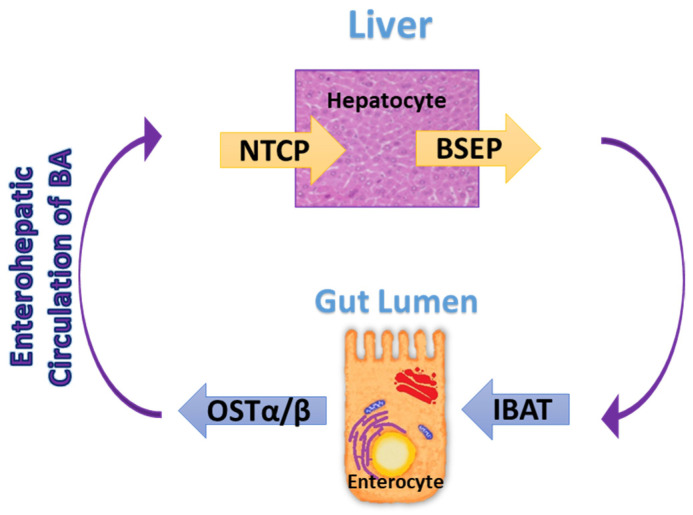
The enterohepatic circulation. BAs are synthesized in hepatocytes and secreted into the bile via BSEP; the bile flows into the gallbladder and then into the duodenum. In the distal ileum, conjugated BAs are absorbed by active transport through the IBAT on enterocytes, where BAs are transported to the basolateral side of the membrane where the OSTα and OSTβ are located, releasing them into the portal circulation, and they enter the enterohepatic bile salt recirculation. Abbreviations: BSEP, bile salt export pump; NTCP, Na+-taurocholate cotransporting polypeptide; OSTα/β, organic solute transporters α and β; IBAT, ileal sodium/bile acid cotransporter.

Inside the enterocyte, BAs are bound to Fatty Acid Binding Protein 6 (FABP6) and transported to the basolateral side of the membrane where the organic solute transporters α and β (OSTα and OSTβ) are located, releasing them into the portal circulation [32]. Via the portal vein, the BAs reach the liver, flow into the sinusoids, and enter the hepatocytes via the sodium/bile acid cotransporter also known as the Na+-taurocholate cotransporting polypeptide (NTCP) (see Figure 4). The enterohepatic circulation is very efficient, reabsorbing about 95% of the BAs (≈2–3 g per cycle for 10 to 12 times a day for a total of ≈20–30 g daily) [54]. Approximately 5% of BAs (≈400–800 mg) escape active absorption and become substrates for colonic bacteria. BSH activity in the small intestine produces unconjugated BAs, which are less polar than conjugated BAs and are inefficiently transported by the ileal sodium/bile acid cotransporter (IBAT) in the ileum. This allows a greater influx of free primary BAs into the colon, where they are metabolized by local bacteria to secondary BAs [30,32]. Secondary BAs can be absorbed by passive transport or excreted in the feces; in the latter case, de novo hepatic biosynthesis from cholesterol replenishes the loss [36]. The enterohepatic circulation is very efficient; low BSH enzyme activity in the elderly with intestinal dysbiosis and/or reduced liver function and/or poor dietary habits may lead to reduced bacterial production of secondary BAs, which, when present at physiological levels, have a protective effect in the colon [30,49,51,64].

### 2.4. Bacterial Bile Acid Metabolism

More than 1/5 of human GM bacteria have the BSH enzyme, which is encoded mainly by bacteria belonging to the phyla Firmicutes, Bacteroidetes, and Actinobacteria [65,66]. BSH activity is present in *Lactobacillus*, *Bifidobacterium*, *Enterococcus*, *Clostridium*, and *Bacteroides* spp. *Lactobacillus* and *Bifidobacteria* can be administered as probiotic strains, whereas *Bacteroides* and *Enterococcus* spp. are only commensal inhabitants of the gastrointestinal tract [52]. Among these bacteria, BSH genes and corresponding enzymes have been identified mainly in bacteria from the human GM, including, but not limited, to *L. salivarius*, *Lactobacillus acidophilus*, *Lactobacillus johnsonii*, *Lactiplantibacillus plantarum*, *Bifidobacterium longum*, *Bifidobacterium bifidum*, *Bifidobacterium adolescentis*, and *Bifidobacterium animalis* [67,68]. Some pathogens such as *Brucella abortus*, *Listeria monocytogenes*, and *Clostridium perfringens* encode BSH [68]. With the exception of *Brucella abortus* and opportunistic pathogens of the *Xanthomonas* genus and two Gram-negative *Bacteroides* strains with BSH activity, all other BSH-positive bacteria are Gram positive [52,68]. Three different classes of BSH have been identified in the genus *Bifidobacterium*, two of which have a high specificity for glycine-conjugated BAs [36]. Human gut archaea species, such as *Methanosphaera stadmanae* and *Methanobrevibacter smithii*, also produce BSH [68].

BSH activity is necessary for the metabolism of deconjugated BAs by other bacteria in the colon, as conjugated BAs are not substrates for 7-dehydroxylation [32]. In fact, deconjugated BAs serve as substrates for the production of secondary BAs [67]. The 7-dehydroxylation of BAs seems to be restricted to a few bacterial families such as Ruminococcaceae, Peptostreptococcaceae, Lachnospiraceae, and Oscillospiraceae [32]. DCA and LCA are produced by *Eubacterium* spp. and by a small community of bacteria belonging to the genus *Clostridium* spp., e.g., *C. sordelli*, *C. hiranonis*, *C. hylemonae*, and *C. scindens* in the colon [30,69]. UDCA is produced by bacteria such as *Ruminococcus gnavus*, *Clostridium absonum* (observed only in culture medium), *Clostridium baratii*, and others not yet well identified [30]. Antibiotics are one of the factors that have the greatest impact on the diversity of microbial species [70]. Antibiotic treatments reduce GM diversity, BSH enzyme activity, and 7α-dehydroxylase enzyme activity, leading to an imbalance in the concentration of circulating BAs by increasing primaries over secondaries [67]. Studies in a mouse model have confirmed that the use of different antibiotics leads to a predominance of primary over secondary BAs in the host and predisposes to *Clostridioides difficile* infection [71,72,73]. The enzymes encoded by the bai operon have evolved to recognize BAs produced endogenously by the host, the only exception being UDCA, which, although taken up exogenously, is recognized as endogenous and converted to LCA by the bacterial enzyme 7β-dehydroxylase [32].

## 3. GM and Bile Acid Relationship in Elderly People

**Figure 5:**
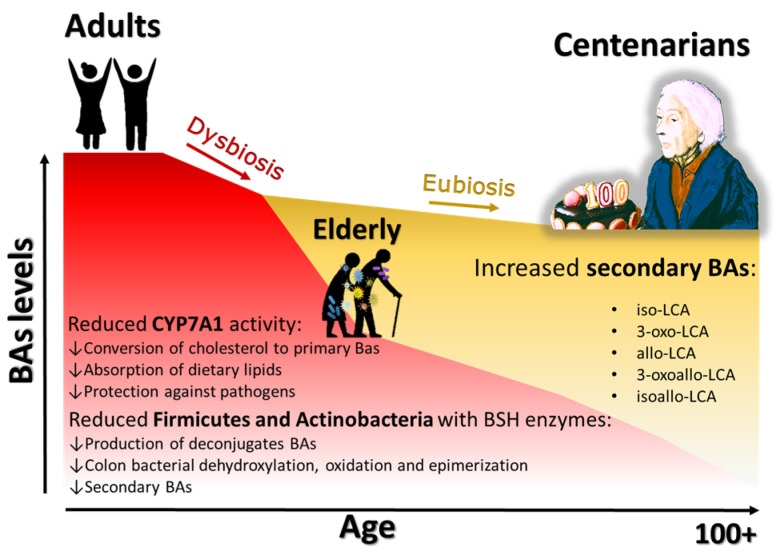
Relationship between GM and BAs in the elderly. During aging, in people with a dysbiosis status of GM, we observe a general reduction in the levels of BAs (red part of the graph). This reduction is accompanied by a decrease in CYP7A1 activity and a reduction in Firmicutes and Actinobacteria. If the dysbiotic status is not well corrected, there are several consequences: a reduction in primary, secondary, and deconjugated BAs, a reduction in the absorption of dietary lipids, and a reduction in the defense against pathogens. Conversely, eubiosis in later life, as in the case of centenarians, is associated with the maintenance of the BA levels, particularly secondary BAs (yellow part of the graph).

In general, it is thought that in elderly people the volume of the liver is reduced by 20–40% due to reduced blood supply, and the capacity for liver regeneration is reduced. However, considering individual differences, liver function remains relatively preserved [74]. CYP7A1 is an enzyme of the cytochrome P-450 family of hepatic monooxygenases that catalyzes the first step in the conversion of cholesterol to BAs in the classical pathway [75]. Reduced activity of this enzyme can diminish the conversion of cholesterol to BAs and the ability to solubilize and absorb dietary lipids through micelles and reduce protective activity against colonization by intestinal pathogens [75]. BA levels change throughout life, and in older people, BA production may be reduced, depending on individual health, diet, and/or other factors [37]. Bertolotti et al. observed that in humans there is an inverse correlation between age and CYP7A1 enzyme expression; in fact, in their study, they reported that CYP7A1 enzyme activity is reduced by 50% in elderly people aged >65 years compared to those aged <65 years [76]. Similar studies have been performed in mice by Wang, who showed that aging itself may be a cause of reduced CYP7A1 enzyme activity, which in turn leads to less de novo synthesis of BAs from cholesterol [77]. After synthesis in the liver, BAs are secreted with the bile, reach the intestine, and are modified by bacterial enzymes [30,53]. In the elderly, the GM changes qualitatively/quantitatively, and the bacteria that have the enzymes capable of metabolizing BAs are reduced [24,78]. The reduction in Firmicutes (in particular the genus *Lactobacillus*) and Actinobacteria (in particular the genus *Bifidobacterium*) equipped with BSH enzymes results in a lower production of deconjugated BAs [24,51,68,78] (see Figure 5).

The smaller amount of deconjugated BAs that reach the colon still serve as a substrate for further bacterial metabolism of dehydroxylation, oxidation, and epimerization to produce secondary BAs but may not be sufficient to prevent colonization and/or local infection by pathogens [11,21,22,23,24,32,34,49,51]. The toxic properties of DCA and LCA are well known [32]. In clinical studies, elevated levels of DCA have been found in the serum and feces of patients with colorectal cancer (CRC). DCA is a BA with pro-tumor properties and is capable of inducing CRC by causing DNA damage to cells and disrupting the continuity of the intestinal mucosal barrier [79].

It is also known that chronic high levels of secondary BAs in the liver can lead to cirrhosis, fibrosis, and organ failure and may even induce hepatocellular carcinoma (HCC) and cholangiocarcinoma [80,81]. However, it has been shown that the accumulation of ≈500 μM DCA and LCA can block the growth of *C. difficile* [82]. Another study has shown that in people with recurrent *C. difficile* infection, there is an increase in primary BAs and a decrease in secondary BAs [30]. In addition, DCA and isoallo-LCA can reduce the virulence of *C. perfringens* by transcriptional modulation of the pathogen’s signaling pathways [83].

LCA showed protective properties in colon cells and anti-inflammatory effects in mouse models of colitis [84]. More generally, studies in mouse models of cholestasis and/or biliary obstruction have highlighted the role of BAs in preventing the colonization of the gut by pathogenic bacteria and their permeation into the bloodstream [85,86].

Increased bacterial proliferation in the digestive tract has also been observed in liver disease or digestive disorders where BA production is impaired [60]. The analysis of BAs in fecal samples from centenarians showed an increase in secondary BAs such as isoLCA, 3-oxo-LCA, allo-LCA, 3-oxoallo-LCA, and isoallo-LCA with respect to older and young people [87,88]. Zhou et al. reported BAs as molecules capable of regulating host longevity [49]. The role of BAs appears to be closely linked to their quantity and quality [32].

Reduced production of secondary BAs and reduced fecal excretion of these acids may contribute to both increased hepatic cholesterol accumulation and elevated serum LDL levels. This may promote a state of intestinal dysbiosis, which in turn may promote intestinal pathogen infection and local inflammation [78] (see Figure 5).

## 4. Interventions Targeting Gut Microbiota to Improve Secondary Bile Acids Levels

### 4.1. UDCA/TUDCA Supplementations

UDCA has antifibrotic, anticholestatic, antiproliferative, and anti-inflammatory properties and is used in cases where BA production is impaired. UDCA supplementation is used to treat a variety of conditions including gallstones, primary biliary cirrhosis, primary sclerosing cholangitis, non-alcoholic fatty liver disease (NAFLD), chronic viral hepatitis C, recurrent colonic adenomas, cholestasis of pregnancy, and recurrent pancreatitis [64,89,90,91,92,93,94,95,96,97]. Digler et al. studied the effect of 15 mg/kg/day of UDCA for 3 weeks on subjects with primary biliary cholangitis and observed a decrease in primary conjugated BAs, but the effects of the treatment on the GM were not investigated [98]. In addition, two meta-analyses showed that treatment with a low dose and a standard dose of UDCA (8–15 mg per kg per day) was associated with a significant reduction in the risk of colorectal neoplasia [99,100,101]. UDCA is partially absorbed in the small intestine, so only a fraction reaches the colon. Furthermore, in the colon, bacteria with bai enzymes do not metabolize exogenously BAs, with the exception of UDCA. UDCA reaches the colon when introduced and can be transformed into LCA [32]. Subsequently, the 3α-OH form of LCA can be converted to the 3β-OH form by 3α-HSDH and 3β-HSDH enzymes; this modification leads to the formation of isoallo-LCA that can directly influence the structure of gut microbial communities and protect against potential multidrug-resistant Gram-positive pathogens, such as *Clostridioides difficile* and *Enterococcus faecium* [32,88].

There is no strong evidence that exogenously administered UDCA has an effect on the structure of the GM, but several authors reported the ability of primary BAs to alter the structure of the GM in mice and rats [4,30,81,102]. In addition, TUDCA can also be used as a supplement in cases of impaired BA metabolism [4,102,103,104]. Also, the administration of a 5 g TUDCA/kg lithogenic diet to mice increased the F/B ratio by 3.13-fold [105]. In our recent case report, we demonstrated that the GM of a 92-year-old woman showed an increase in Firmicutes (including Ruminococcaceae), which was probably due to prolonged use of TUDCA [103]. We hypothesized a role for TUDCA in increasing the proportion of Firmicutes by increasing the number of BAs in the colon.

### 4.2. Probiotics Supplementations

In the elderly, the presence of *Lactobacillus* and *Bifidobacterium* is reduced, so supplementation of these bacteria may be useful [23,24,26,76]. Furthermore, in the elderly, BSH activity and reduced fecal excretion of secondary BAs may contribute to both increased hepatic cholesterol accumulation and elevated serum LDL levels. This may promote a state of intestinal dysbiosis, which in turn may promote intestinal pathogen infection and local inflammation [76]. A study in mice showed that supplementation with a probiotic consisting of *Bifidobacterium breve*, *Bifidobacterium longum*, *Bifidobacterium infantis*, *Lactobacillus acidophilus*, *Lactiplantibacillus plantarum*, *Lacticaseibacillus paracasei*, *Lactobacillus delbrueckii* subsp. *bulgaricus*, and *Streptococcus thermophiles* at a dose of 50 × 10⁹ CFU/day for 21 days increased the concentration of deconjugated BAs [47]. The beneficial effects of *Lactobacillus* and *Bifidobacterium* supplementation are well discussed in the review by Sivamaruthi et al. [51]. The increased colonization of Lactobacilli and Bifidobacteria in the gut promotes an increased production of deconjugated BAs and a subsequent increase of de novo synthesis of BAs from cholesterol to compensate for the loss of BAs in the feces [51]. Oral administration of *Bifidobacterium longum* SPM1207 (10^8^~10^9^ CFU/mL daily for two weeks) increased fecal excretion of BAs [51,106]. Interestingly, *Bifidobacterium longum* W11 has been described as a strain resistant to the antibiotic rifaximin, which is commonly used in the clinic for various purposes. Normally, other strains of Bifidobacteria do not survive antibiotic treatment [107]. In addition to Lactobacilli and Bifidobacteria, it has been suggested that other types of bacteria may influence BAs and cholesterol concentrations [107]. A study in rats showed that feeding a 30 g rice bran/kg diet fermented with *Bacilli*, *Lactobacilli*, *Streptococci*, *Clostridium butyricum*, *Saccharomyces cerevisiae*, and *Candida utilis* significantly reduced cholesterol concentrations [108]. These include *Clostridium butyricum*, a bacterium that is commercially available as a probiotic, and when it is given during *C. difficile* infection, it can play an important role in reducing the damage to the intestinal epithelium caused by the pathogen [109]. In addition to lowering serum cholesterol levels, the use of probiotics can restore a state of intestinal eubiosis [51].

### 4.3. Dietary Intervention

A Mediterranean diet rich in fiber (33 g per day), complex carbohydrates (such as 3–9 servings of vegetables, 0.5–2 servings of fruit, and 1–13 servings of cereals), and olive oil (up to 8 servings) promotes the growth of *Bifidobacterium longum* and *Bifidobacterium breve* [110]. Furthermore, the soluble component of complex carbohydrates allows for the development of butyrate-producing bacteria, such as *Clostridium leptum* and *Eubacterium rectale* [110]. Butyrate-producing bacteria, such as *Eubacterium* spp., are able to transform primary BAs into DCA and LCA [69]. Increased fiber intake raises BA excretion over 24 h after ingestion [110]. Increased inulin-type fructan (ITF) prebiotic (inulin/oligofructose 50/50, 16 g per day [111]) intake also increased levels of *Bifidobacteria* and *Faecalibacterium prausnitzii* [112]. Interestingly, a very recent paper has evidenced that *F. prausnitzii* is capable of counteracting the Enterobacteriaceae colonization of GM [113]. A study has demonstrated that fiber-rich vegan (200 g of inulin) and omnivore (50 g of cellulose) diets were associated with a rapid recovery of BSH activity after antibiotic treatment, indicating a positive association between dietary fibers and BSH activity [114]. Conversely, David et al. described that a 5-day animal-based diet (136.8 g of fat, 126.5 g of protein, 2.7 g of carbs, and 0.0 g of fiber) increased BSH expression and increased fecal BA concentration [115]. An analysis of the feces of the vegan subjects showed a significant reduction in the fecal LCA compared to the omnivorous subjects [116]. In addition to fibers, the proportion of lipids in the diet can also influence BA levels, with high-fat diets (100 g of protein, 249 g of fat, 14 g of carbs, 300 mg of cholesterol, and 15 g of fiber) increasing CA levels and low-fat diets (100 g of proteins, 1 g of fat, 572 g of carbs, 300 mg of cholesterol, and 15 g of fiber) reducing them [46]. Salonen et al. reported a strong individuality in response to diet, the magnitude of which appears to depend on the initial composition of the GM [112]. In general, diets low in protein and fat or high in carbohydrates and fiber are able to regulate BSH and BA levels in a way that provides the benefits of secondary BAs without side effects.

## 5. Conclusions

The interaction between GM and BAs needs to be further explored and studied in depth. In particular, much remains to be learned about secondary BAs, which have been shown to play a role in promoting healthy aging. Indeed, the paper by Sato et al. [88] demonstrated a shift towards a unique profile in the composition of GM in centenarians. The GM bacteria of centenarians are enriched in unique taxa capable of producing secondary BAs. Accordingly, this uniqueness is defined as a hallmark of aging by Lopez et al. [117]. Much remains to be learned about secondary BAs. What is known is that GM-derived secondary BAs have multiple and often conflicting roles, with both beneficial and detrimental effects on host health. In recent years, attention to the GM has increased exponentially, leading to many discoveries. A very recent and interesting paper looking at the GM of over 12,000 people found that certain bacteria, such as *F. prausnitzii*, are able to contain potentially pathogenic infections from *E. coli* and *K. pneumoniae* [113]. The authors believe that even through a healthy diet, our bacteria could be natural antibiotics. In the CONSORTIUM phase 2 study, the use of VE303, a consortium of eight bacterial strains, was able to prevent the recurrence of *Clostridioides difficile* infection (CDI) by also increasing the secondary BAs, such as UDCA [118]. Given the exponential growth of antibiotic resistance and the general aging of the population, the ability to maintain a GM in eubiosis could be a guarantee of well being and improved quality of life. We recently observed that TUDCA was able to alter the structure of the GM in a pro-Firmicutes key in an elderly patient fed home enteral nutrition based on an oligomeric mixture [103]. In general, Firmicutes bacteria tend to decline with age. In this review, we reported literature data on three types of interventions (diet, UDCA/TUDCA, and probiotic supplementation) that were able to restore BSH activity and derive metabolites and may be effective in the elderly.

## Figures and Tables

**Table 1:** Diversity of the known human BAs built off the same sterol backbone with variations in hydroxylated positions, presence of ketones, and substitution groups “R”.

Name	Abbreviation	R1	R2	R3	R4
Chenodeoxycholic acid	CDCA	-OH (α)	-OH (α)	-H	-OH
Ursodeoxycholic acid	UDCA	-OH (α)	-OH (β)	-H	-OH
Isochenodeoxycholic acid	iCDCA	-OH (β)	-OH (α)	-H	-OH
Cholic acid	CA	-OH (α)	-OH (α)	-OH (α)	-OH
12-epicholic acid	12-EPA	-OH (α)	-OH (α)	-OH (β)	-OH
Ursocholic acid	UCA	-OH (α)	-OH (β)	-OH (α)	-OH
Isocholic acid	ICA	-OH (β)	-OH (α)	-OH (α)	-OH
Lithocholic acid	LCA	-OH (α)	-H	-H	-OH
Isolithocholic acid	isoLCA	-OH (β)	-H	-H	-OH
Deoxycholic acid	DCA	-OH (α)	-H	-OH (α)	-OH
Isodeoxycholic acid	isoDCA	-OH (β)	-H	-OH (α)	-OH
Lithocholic acid 3-sulfate	3-sulfo-LCA	-OSO_3_H	-H	-H	-OH
Glycocholic acid	GCA	-OH (α)	-OH (α)	-OH (α)	-NHCH_2_COO-
Glycochenodeoxycholic acid	GCDCA	-OH (α)	-OH (α)	-H	-NHCH_2_COO-
Taurocholic acid	TCA	-OH (α)	-OH (α)	-OH (α)	-NHCH_2_CH_2_SO_3_-
Taurochenodeoxycholic acid	TCDCA	-OH (α)	-OH (α)	-H	-NHCH_2_CH_2_SO_3_-
Tauroursodeoxycholic acid	TUDCA	-OH (α)	-OH (β)	-H	-NHCH_2_CH_2_SO_3_-
Dehydrolithocholic acid	3-oxo-LCA	-O	-H	-H	-OH
3α-hydroxy-5α-cholanoic acid	allo-LCA	-OH (α)	-H	-H	-OH
3-oxoallolithocholic acid	3-oxoallo-LCA	-O	-H	-H	-OH
Isoallolithocholic acid	isoallo-LCA	-OH (β)	-H	-OH (α)	-OH
